# Glycine ethyl ester hydro­chloride

**DOI:** 10.1107/S1600536810028849

**Published:** 2010-07-24

**Authors:** Yong-Jun He, Pei Zou, Hong-Yong Wang, Hao Wu, Min-Hao Xie

**Affiliations:** a Jiangsu Institute of Nuclear Medicine, Wuxi 214063, People’s Republic of China

## Abstract

In the crystal structure of the title compound, C_4_H_10_NO_2_^+^·Cl^−^ (systematic name: 3-eth­oxy-3-oxopropan-1-aminium chlor­ide), there are strong inter­molecular N—H⋯Cl, C—H⋯Cl and C—H⋯O hydrogen-bonding inter­actions between the free chloride anion and the organic cation, resulting in a two-dimensional supra­molecular network in the *ab* plane.

## Related literature

The title compound is an inter­mediate in the synthesis of dichloro­vinyl­cyclo­propane carb­oxy­lic acid, see: Xue (1995 ▶). For related structures, see: Taubald *et al.* (1984 ▶); Gainsford *et al.* (1986 ▶); Eduok *et al.* (1994 ▶).
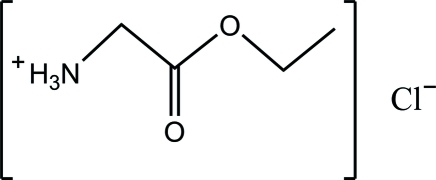


## Experimental

### 

#### Crystal data

C_4_H_10_NO_2_^+^·Cl^−^
*M* *_r_* = 139.58
Monoclinic, 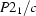

*a* = 8.965 (3) Å
*b* = 12.543 (4) Å
*c* = 5.972 (2) Å
β = 103.630 (5)°
*V* = 652.6 (4) Å^3^
*Z* = 4
Mo *K*α radiation
μ = 0.50 mm^−1^
*T* = 123 K
0.33 × 0.33 × 0.23 mm

#### Data collection

Rigaku SPIDER diffractometer
4996 measured reflections
1489 independent reflections
1294 reflections with *I* > 2σ(*I*)
*R*_int_ = 0.024

#### Refinement

*R*[*F* ^2^ > 2σ(*F* ^2^)] = 0.027
*wR*(*F* ^2^) = 0.064
*S* = 1.00
1489 reflections
87 parameters
H atoms treated by a mixture of independent and constrained refinement
Δρ_max_ = 0.40 e Å^−3^
Δρ_min_ = −0.21 e Å^−3^

### 

Data collection: *RAPID-AUTO* (Rigaku, 2004 ▶); cell refinement: *RAPID-AUTO*; data reduction: *RAPID-AUTO*; program(s) used to solve structure: *SHELXS97* (Sheldrick, 2008 ▶); program(s) used to refine structure: *SHELXL97* (Sheldrick, 2008 ▶); molecular graphics: *SHELXTL* (Sheldrick, 2008 ▶); software used to prepare material for publication: *SHELXTL*.

## Supplementary Material

Crystal structure: contains datablocks I, global. DOI: 10.1107/S1600536810028849/bv2143sup1.cif

Structure factors: contains datablocks I. DOI: 10.1107/S1600536810028849/bv2143Isup2.hkl

Additional supplementary materials:  crystallographic information; 3D view; checkCIF report

## Figures and Tables

**Table 1:** Hydrogen-bond geometry (Å, °)

*D*—H⋯*A*	*D*—H	H⋯*A*	*D*⋯*A*	*D*—H⋯*A*
N1—H0*A*⋯Cl1	0.904 (17)	2.300 (17)	3.1845 (16)	166.1 (12)
N1—H0*B*⋯Cl1^i^	0.906 (18)	2.386 (18)	3.1658 (16)	144.3 (15)
N1—H0*C*⋯Cl1	0.890 (19)	2.435 (19)	3.2566 (16)	153.7 (15)
C1—H1*A*⋯O2	0.99	2.47	2.9072 (18)	106
C3—H3*B*⋯Cl1^ii^	0.99	2.79	3.7529 (18)	164

Symmetry codes: (i) 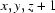; (ii) 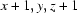.

**Fig. 1.**
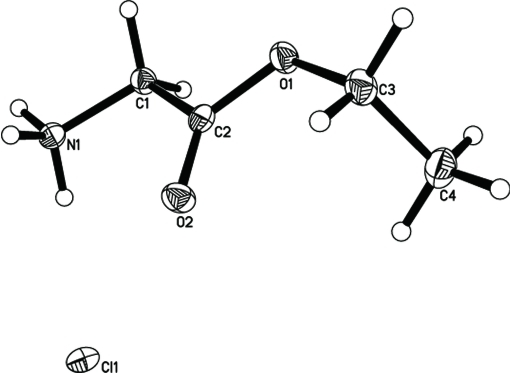
A view of the title compound with the atomic numbering scheme. Displacement ellipsoids were drawn at the 50% probability level.

**Fig. 2.**
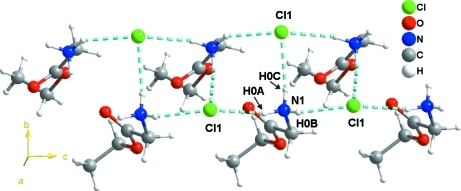
A view of the packing arrangement of the title compound. Hydogran bonds are shown by dashed lines.

## supplementary crystallographic information

### Comment

The title compound, glycine ethyl ester hydrochloride is used in the preparation of dichlorovinylcyclopropane carboxylic acid, an important pesticide intermediate (Xue,1995).It is also used in the preparation of function material, the crystal structures of dichloro-bis(glycine ethyl ester)-palladium(II) (Taubald,*et al.*, 1984),*p,p*-(µ_2_-peroxo) -bis(tris(2-aminoethyl)-amine-*N*,*N*',*N*'',*N*''')-bis(ethylglycinate-N)-cobalt(II) tetraperchlorate (Gainsford *et al.*, 1986),cis-β_2_-((s,s)-chloro-(glycine ethyl ester-*N*)-(triethylenetetramine)-cobalt(III) dichloride trihydrate (Eduok *et al.*, 1994) have been reported. The molecular structure of(I) is shown in Fig.1. The three crystallographically independent N—H moieties are engaged in highly directional N^+^—H···Cl^-^ hydrogen bonds with three symmetry-related Cl^-^ anions. These interactions promote the formation of a tape of C_4_H_10_NO_2_^+^.Cl^-^ moieties running parallel to the c axis.

### Experimental

Glycine ethyl ester hydrochloride (0.1 mmol, Sigma Aldrich at 99% purity) was dissolved methanol (20 ml) and gently heated under reflux for 1 h. After cooling the solution to ambient temperature, crystals suitable for single-crystal X-ray diffraction were grown by slow evaporation of the solvent after few days.

### Refinement

Hydrogen atoms bound to nitrogen and carbon were located at their idealized positions and were included in the final structural model in riding-motion approximation with C—H = 0.98Å and N—H = 0.90 Å. The isotropic thermal displacement parameters for these atoms were fixed at 1.2 (for the -CH_2_- and -CH_3_ group) or 1.5 (for the pendant -NH_3_^+^ moieties) times U_eq_ of the atom to which they are attached.

### Crystal data

**Table:**

C_4_H_10_NO_2_^+^·Cl^−^	*F*(000) = 296
*M**_r_* = 139.58	*D*_x_ = 1.421 Mg m^−^^3^
Monoclinic, *P*2_1_/*c*	Melting point: 145(1) K
Hall symbol: -P 2ybc	Mo *K*α radiation, λ = 0.71073 Å
*a* = 8.965 (3) Å	Cell parameters from 1964 reflections
*b* = 12.543 (4) Å	θ = 3.3–27.5°
*c* = 5.972 (2) Å	µ = 0.50 mm^−^^1^
β = 103.630 (5)°	*T* = 123 K
*V* = 652.6 (4) Å^3^	Block, colorless
*Z* = 4	0.33 × 0.33 × 0.23 mm

### Data collection

**Table:**

Rigaku SPIDER diffractometer	1294 reflections with *I* > 2σ(*I*)
Radiation source: Rotating Anode	*R*_int_ = 0.024
graphite	θ_max_ = 27.5°, θ_min_ = 3.3°
ω scans	*h* = −10→11
4996 measured reflections	*k* = −16→11
1489 independent reflections	*l* = −7→7

### Refinement

**Table:**

Refinement on *F*^2^	Secondary atom site location: difference Fourier map
Least-squares matrix: full	Hydrogen site location: inferred from neighbouring sites
*R*[*F*^2^ > 2σ(*F*^2^)] = 0.027	H atoms treated by a mixture of independent and constrained refinement
*wR*(*F*^2^) = 0.064	*w* = 1/[σ^2^(*F*_o_^2^) + (0.031*P*)^2^ + 0.160*P*] where *P* = (*F*_o_^2^ + 2*F*_c_^2^)/3
*S* = 1.00	(Δ/σ)_max_ < 0.001
1489 reflections	Δρ_max_ = 0.40 e Å^−^^3^
87 parameters	Δρ_min_ = −0.21 e Å^−^^3^
0 restraints	Extinction correction: *SHELXL97* (Sheldrick, 2008), Fc^*^=kFc[1+0.001xFc^2^λ^3^/sin(2θ)]^-1/4^
Primary atom site location: structure-invariant direct methods	Extinction coefficient: 0.011 (3)

### Special details

**Table:**

Geometry. All esds (except the esd in the dihedral angle between two l.s. planes) are estimated using the full covariance matrix. The cell esds are taken into account individually in the estimation of esds in distances, angles and torsion angles; correlations between esds in cell parameters are only used when they are defined by crystal symmetry. An approximate (isotropic) treatment of cell esds is used for estimating esds involving l.s. planes.
Refinement. Refinement of *F*^2^ against ALL reflections. The weighted *R*-factor wR and goodness of fit *S* are based on *F*^2^, conventional *R*-factors *R* are based on *F*, with *F* set to zero for negative *F*^2^. The threshold expression of *F*^2^ > σ(*F*^2^) is used only for calculating *R*-factors(gt) etc. and is not relevant to the choice of reflections for refinement. *R*-factors based on *F*^2^ are statistically about twice as large as those based on *F*, and *R*- factors based on ALL data will be even larger.

### Fractional atomic coordinates and isotropic or equivalent isotropic displacement parameters (Å^2^)

**Table:**

	*x*	*y*	*z*	*U*_iso_*/*U*_eq_	
Cl1	−0.00205 (3)	0.38254 (2)	0.24012 (5)	0.01640 (11)	
O1	0.52878 (10)	0.38513 (7)	0.85715 (16)	0.0168 (2)	
O2	0.34886 (10)	0.29775 (7)	0.59414 (15)	0.0171 (2)	
N1	0.11868 (13)	0.36318 (9)	0.7845 (2)	0.0144 (2)	
C2	0.38589 (14)	0.35635 (9)	0.7575 (2)	0.0132 (3)	
C1	0.27318 (14)	0.40847 (10)	0.8745 (2)	0.0136 (3)	
H1A	0.3056	0.3965	1.0429	0.016*	
H1B	0.2709	0.4863	0.8461	0.016*	
C3	0.64973 (15)	0.34018 (11)	0.7579 (2)	0.0184 (3)	
H3A	0.6205	0.2672	0.7010	0.022*	
H3B	0.7464	0.3354	0.8786	0.022*	
C4	0.67496 (16)	0.40810 (11)	0.5624 (2)	0.0222 (3)	
H4A	0.5809	0.4096	0.4392	0.027*	
H4B	0.7589	0.3782	0.5029	0.027*	
H4C	0.7015	0.4808	0.6179	0.027*	
H0A	0.0807 (19)	0.3806 (11)	0.635 (3)	0.022 (4)*	
H0B	0.054 (2)	0.3873 (13)	0.869 (3)	0.035 (5)*	
H0C	0.1184 (19)	0.2925 (15)	0.797 (3)	0.033 (5)*	

### Atomic displacement parameters (Å^2^)

**Table:**

	*U*^11^	*U*^22^	*U*^33^	*U*^12^	*U*^13^	*U*^23^
Cl1	0.01827 (17)	0.01928 (19)	0.01196 (16)	0.00511 (12)	0.00419 (11)	0.00078 (11)
O1	0.0131 (4)	0.0206 (5)	0.0171 (5)	−0.0019 (4)	0.0045 (4)	−0.0039 (4)
O2	0.0163 (4)	0.0191 (5)	0.0157 (5)	−0.0003 (4)	0.0033 (4)	−0.0049 (4)
N1	0.0149 (5)	0.0166 (6)	0.0128 (5)	−0.0010 (4)	0.0055 (4)	−0.0022 (4)
C2	0.0150 (6)	0.0120 (6)	0.0131 (6)	−0.0006 (5)	0.0043 (5)	0.0027 (4)
C1	0.0136 (6)	0.0132 (6)	0.0141 (6)	−0.0010 (5)	0.0038 (5)	−0.0020 (5)
C3	0.0130 (6)	0.0224 (7)	0.0202 (7)	0.0013 (5)	0.0046 (5)	−0.0017 (5)
C4	0.0216 (7)	0.0228 (7)	0.0252 (7)	−0.0032 (5)	0.0115 (6)	−0.0029 (6)

### Geometric parameters (Å, °)

**Table:**

O1—C2	1.3290 (15)	C1—H1A	0.9900
O1—C3	1.4654 (16)	C1—H1B	0.9900
O2—C2	1.2040 (15)	C3—C4	1.505 (2)
N1—C1	1.4762 (16)	C3—H3A	0.9900
N1—H0A	0.902 (17)	C3—H3B	0.9900
N1—H0B	0.906 (19)	C4—H4A	0.9800
N1—H0C	0.890 (18)	C4—H4B	0.9800
C2—C1	1.5065 (18)	C4—H4C	0.9800
			
C2—O1—C3	116.20 (10)	C2—C1—H1B	109.7
C1—N1—H0A	111.7 (10)	H1A—C1—H1B	108.2
C1—N1—H0B	109.8 (12)	O1—C3—C4	110.89 (11)
H0A—N1—H0B	109.0 (16)	O1—C3—H3A	109.5
C1—N1—H0C	111.9 (11)	C4—C3—H3A	109.5
H0A—N1—H0C	108.6 (14)	O1—C3—H3B	109.5
H0B—N1—H0C	105.6 (15)	C4—C3—H3B	109.5
O2—C2—O1	125.54 (12)	H3A—C3—H3B	108.0
O2—C2—C1	123.62 (12)	C3—C4—H4A	109.5
O1—C2—C1	110.83 (11)	C3—C4—H4B	109.5
N1—C1—C2	109.79 (10)	H4A—C4—H4B	109.5
N1—C1—H1A	109.7	C3—C4—H4C	109.5
C2—C1—H1A	109.7	H4A—C4—H4C	109.5
N1—C1—H1B	109.7	H4B—C4—H4C	109.5
			
C3—O1—C2—O2	−0.45 (18)	O1—C2—C1—N1	−171.55 (10)
C3—O1—C2—C1	−179.62 (10)	C2—O1—C3—C4	86.87 (14)
O2—C2—C1—N1	9.27 (17)		

### Hydrogen-bond geometry (Å, °)

**Table:**

*D*—H···*A*	*D*—H	H···*A*	*D*···*A*	*D*—H···*A*
N1—H0A···Cl1	0.904 (17)	2.300 (17)	3.1845 (16)	166.1 (12)
N1—H0B···Cl1^i^	0.906 (18)	2.386 (18)	3.1658 (16)	144.3 (15)
N1—H0C···Cl1^ii^	0.890 (19)	2.435 (19)	3.2566 (16)	153.7 (15)
C1—H1A···O2^ii^	0.99	2.47	2.9072 (18)	106
C3—H3B···Cl1^iii^	0.99	2.79	3.7529 (18)	164

Symmetry codes: (i) *x*, *y*, *z*+1; (ii) ; (iii) *x*+1, *y*, *z*+1.
